# Aberrant *MET* Receptor Tyrosine Kinase Signaling in Glioblastoma: Targeted Therapy and Future Directions

**DOI:** 10.3390/cells13030218

**Published:** 2024-01-25

**Authors:** Abdulhameed Al-Ghabkari, Bruce Huang, Morag Park

**Affiliations:** 1 Rosalind and Morris Goodman Cancer Institute, McGill University, Montreal, QC H3A 1A3, Canada; abdulhameed.alghabkari@mcgill.ca (A.A.-G.); bruce.huang@mail.mcgill.ca (B.H.); 2 Department of Biochemistry, McGill University, Montreal, QC H3G 1Y6, Canada; 3 Department of Oncology, McGill University, Montreal, QC H4A 3T2, Canada; 4 Department of Medicine, McGill University, Montreal, QC H4A 3J1, Canada

**Keywords:** glioblastoma (GBM), hepatocyte growth factor/scatter factor (HGF/SF), *MET*-targeted therapies, *MET* exon 14 skipping

## Abstract

Brain tumors represent a heterogeneous group of neoplasms characterized by a high degree of aggressiveness and a poor prognosis. Despite recent therapeutic advances, the treatment of brain tumors, including glioblastoma (GBM), an aggressive primary brain tumor associated with poor prognosis and resistance to therapy, remains a significant challenge. Receptor tyrosine kinases (RTKs) are critical during development and in adulthood. Dysregulation of RTKs through activating mutations and gene amplification contributes to many human cancers and provides attractive therapeutic targets for treatment. Under physiological conditions, the Met RTK, the hepatocyte growth factor/scatter factor (HGF/SF) receptor, promotes fundamental signaling cascades that modulate epithelial-to-mesenchymal transition (EMT) involved in tissue repair and embryogenesis. In cancer, increased Met activity promotes tumor growth and metastasis by providing signals for proliferation, survival, and migration/invasion. Recent clinical genomic studies have unveiled multiple mechanisms by which *MET* is genetically altered in GBM, including focal amplification, chromosomal rearrangements generating gene fusions, and a splicing variant mutation (exon 14 skipping, METex14del). Notably, *MET* overexpression contributes to chemotherapy resistance in GBM by promoting the survival of cancer stem-like cells. This is linked to distinctive Met-induced pathways, such as the upregulation of DNA repair mechanisms, which can protect tumor cells from the cytotoxic effects of chemotherapy. The development of *MET*-targeted therapies represents a major step forward in the treatment of brain tumours. Preclinical studies have shown that *MET*-targeted therapies (monoclonal antibodies or small molecule inhibitors) can suppress growth and invasion, enhancing the efficacy of conventional therapies. Early-phase clinical trials have demonstrated promising results with *MET*-targeted therapies in improving overall survival for patients with recurrent GBM. However, challenges remain, including the need for patient stratification, the optimization of treatment regimens, and the identification of mechanisms of resistance. This review aims to highlight the current understanding of mechanisms underlying *MET* dysregulation in GBM. In addition, it will focus on the ongoing preclinical and clinical assessment of therapies targeting *MET* dysregulation in GBM.

## 1. Introduction

GBM is the most common and most aggressive type of brain malignancy in adults and is generally characterized by poor survival, with a median survival of less than one year from diagnosis [1]. Although there has been a significant improvement in overall survival rates of various types of malignant brain tumors over the past decade, the prognosis for GBM patients has exhibited a persistent and concerning lack of progress, with consistently low survival rates [2,3]. Although advancements have been achieved in the current standard of care, which encompasses surgical interventions, radiotherapy, and chemotherapy and mainly temozolomide (TMZ), a DNA alkylating agent that was first approved for medical use in Europe and the United States in the early 2000s, they have sadly failed to improve the prognosis for GBM patients [4,5]. 

GBMs are malignant tumors that arise from glial cells and are classified as high-grade gliomas according to the histological criteria outlined by the World Health Organization (WHO), defined by the presence of either microvascular proliferation or tumor necrosis [6,7]. GBMs are classified as primary or secondary based on their origin and development. Primary GBMs arise de novo, whereas secondary GBMs evolve from pre-existing low-grade astrocytomas. Primary GBMs constitute the majority of cases, accounting for approximately 90%, and are characterized by rapid and spontaneous expansion without evidence of less malignant precursor tumors [7]. Primary GBMs predominantly affect elderly patients and exhibit a significantly poorer prognosis compared to secondary GBMs. Secondary GBMs emerge from grade II and III astrocytomas, oligodendrogliomas, or oligoastrocytomas and are relatively less common, accounting for 5 to 10% of cases and typically manifesting in younger individuals [8,9,10]. The gold-standard treatment for newly diagnosed patients includes maximum safe surgical resection followed by radiotherapy with concurrent and adjuvant chemotherapy, with or without tumor treating fields (TTFields). Despite the ever-growing array of emerging biologics and immunotherapeutic approaches in the field of cancer treatment, it is noteworthy that temozolomide remains the sole systemic therapy that has shown tangible improvements in GBM survival outcomes [11,12]. Considering the inadequate efficacy demonstrated by the currently approved treatment options for GBM, an urgent necessity exists for the development of novel therapeutic strategies. Various biological impediments, including the blood–brain barrier (BBB), the tumor and immune microenvironment, and the presence of intratumor heterogeneity (ITH), have hampered the advancement of novel therapeutic interventions for GBM. These factors present substantial challenges in developing innovative treatment modalities [13]. In particular, a study by Patel et al. using single-cell RNA sequencing revealed that a heterogeneous mixture of cells representing different GBM subgroups could coexist within a single tumor [14]. The findings of this study revealed a compelling association between increased heterogeneity and unfavourable survival outcomes in GBM patients. These findings indicate that the clinical prognosis of proneural GBM is influenced by the relative abundance of tumor cells belonging to alternative subtypes, thereby highlighting the notable clinical relevance of ITH. The advancement of recent technologies has facilitated more comprehensive genetic and epigenetic landscape analyses on larger glioma sample cohorts, enabling the discovery of numerous significant findings in recent years. Among these discoveries, one of the most remarkable and clinically significant observations is the prevalence of mutations in the genes isocitrate dehydrogenase 1 and 2 (IDH1 and IDH2) within a substantial proportion of lower-grade gliomas. Accumulating evidence suggests that these mutations play a causal role in gliomagenesis, profoundly influence tumor biology, and hold clinical and prognostic implications [15]. An in-depth investigation into the molecular aberrations of GBMs has unveiled a broad spectrum of chromosomal alterations. These encompass amplifications of RTK, including chromosome 4 (*PDGFRA*), chromosome 7 (*EGFR*, *MET*), and other genes, including CDK6 and chromosome 12 (*CDK4*, *MDM2*), as well as deletions in chromosome 10, contributing to the loss of a tumor suppressor (*PTEN*) [16]. In addition, somatic genome alterations demonstrate higher frequency in TP53 (34.4%), *EGFR* (32.6%), *PTEN* (32%), tumor suppressor *NF1* (neurofibromin 1, 13.7%), and lipid kinase *PIK3CA* (12%) [17]. Genome-wide methylation analysis of GBM has revealed biologically distinctive DNA methylation events within the promoter region of the MGMT (O6-methyl guanine DNA methyltransferase) gene, as well as CD81, which is involved in DNA repair and radioresistance in a substantial proportion of GBM patients. These distinctive methylation patterns are frequently observed, indicating their potential significance in GBM pathogenesis. Additionally, other key genes, such as *GATA6* (GATA binding protein 6), *DR4* (death receptor 4), and *CASP8* (caspase-8), which are involved in cell adhesion, apoptosis, and proliferation, respectively, exhibit prominent methylation alterations, further emphasizing their potential role in the molecular landscape of GBM subsets [18,19]. A deep understanding of these complex genomic alterations is crucial for uncovering the basic mechanisms underlying GBM development. This comprehensive understanding not only enhances our knowledge of disease pathogenesis but also holds the potential to provide invaluable insights into the design and development of precise and targeted therapeutic strategies. 

RTKs are transmembrane cell-surface proteins that act as signal transducers and mediate key roles in regulating various cellular processes during embryogenesis and in adulthood, such as control of cell growth, survival, differentiation, metabolism, and cell migration and invasion [20,21,22]. The dysregulation of RTK signaling, often caused by gain-of-function alterations, leads to developmental abnormalities and is implicated in a wide range of cancers. Although RTKs function as central regulators of normal cellular processes, the dysregulation of growth factor signaling pathways via genomic alterations has been identified as a key event in human GBMs, and approximately 86% of these tumors harbour at least one genetic event in the core RTK/PI3K pathway [23,24]. In this regard, the initial studies on ITH in GBM revealed the coactivation of multiple RTKs, including EGFR, *MET*, and PDGFR, necessitating a poly-targeting approach to disrupt downstream signaling pathways [25,26]. These findings highlight the complex interplay of RTK signaling in GBM and the need for comprehensive strategies targeting multiple RTKs to modulate downstream signaling pathways and combat tumor progression effectively. Accumulating evidence highlights the significant involvement of *MET* in pivotal aspects of glioma cell biology, including tumor proliferation, growth, migration, invasion, angiogenesis, and stemness [23,27,28]. 

This comprehensive review examines the current knowledge regarding the aberrations observed within Met signaling pathways in GBMs. We also assess the potential of targeted therapies based on insights derived from preclinical and clinical investigations. We will explore various avenues of research and shed light on recent advancements in *MET*-targeted therapies, elucidating their significance in the quest for more effective treatment approaches to manage GBM.

## 2. Met Structure and Function

**Figure 1:**
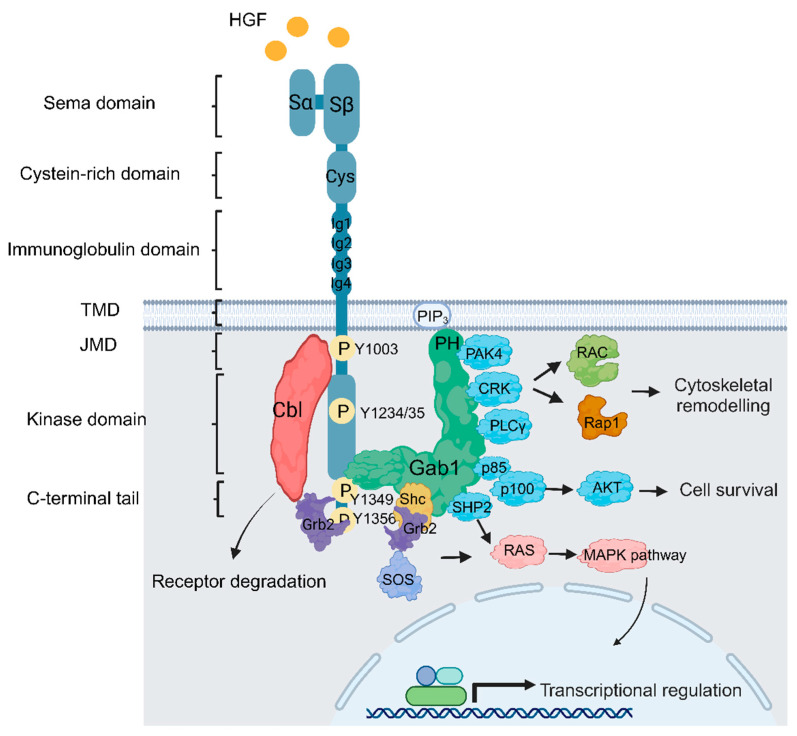
Met structure and function. Following HGF binding to *MET* triggers *MET* dimerization (Met is illustrated as monomer to simplify the model) and phosphorylation of Met within the activation loops, leading to activation of the receptor and followed by subsequent phosphorylation events in the c-terminal domain (Y1349 and Y1356) that enables Met to engage with a number of downstream signaling pathways, such as cytoskeletal remodelling, cell proliferation, and cell survival through coupling Met with multiple adaptor proteins, such as growth factor receptor-bound protein 2 (Grb2), Src homology 2 domain-containing (Shc), and the p85 subunit of phosphoinositide 3-kinases (PI3K). In addition, Grb2 recruits the docking protein Grb2-associated-binding protein 1 (Gab1), which can recruit other key signaling elements, such as tyrosine phosphatase SRC homology 2 domain-containing phosphatase 2 (SHP2), CRK, and PAK4. Additionally, Grb2 serves the crucial function of recruiting the c-Cbl ubiquitin ligase, which acts as a negative regulator of Met.

The Met RTK was first identified as a chromosomal rearrangement induced by exposure to the carcinogen N-methyl-N′-nitronitrosoguanidine in a human osteogenic sarcoma cell line. This event resulted in the formation of a fusion protein known as Tpr-Met, wherein a leucine zipper dimerization domain was fused with the Met cytoplasmic domain. Consequently, this structural alteration led to the constitutive activation of the kinase domain [29,30,31,32]. This groundbreaking discovery sheds light on the role of RTK fusions and Met in oncogenic activities. The Met receptor is a single-pass transmembrane protein. The Met extracellular domain comprises four immunoglobulin-like (Ig-like) domains, a sema domain critical for binding hepatocyte growth factor (HGF), as well as a heparin-binding domain that enhances biological response [33]. The intracellular domain contains a tyrosine kinase domain, activated following ligand binding, which results in tyrosine phosphorylation of tyrosine residues within the kinase domain, as well as a juxtamembrane domain and a carboxy tail, which act as substrate binding sites and promote downstream signaling pathways (Figure 1) [34,35,36].

HGF plays a vital role in maintaining normal tissue homeostasis and is primarily synthesized in the liver. Its expression is upregulated during liver regeneration, particularly by Kupffer cells, and Met-HGF signaling is critical for full liver regeneration [37,38,39]. The HGF gene is located on chromosome 7q21.1; the protein consists of six distinct structural domains, including a short N-terminal domain, four kringle domains (K1-K4), and a non-catalytic serine proteinase homology (SPH) domain. HGF is initially secreted as an inactive precursor (pro-HGF) and subsequently activated through proteolytic processing in the extracellular environment. Processing leads to the formation of mature HGF, which exists as a heterodimer composed of a 69 kDa alpha chain and a 34 kDa beta chain, linked together by a single disulfide bond [40]. The HGF-Met interaction promotes dimerization and potentially oligomerization of the Met receptor and subsequent trans-phosphorylation of tyrosine residues Y1234 and Y1235 within its kinase domain [41]. This phosphorylation event initiates the trans-phosphorylation of tyrosine residues (Y1349, Y1356) located in the C-terminal tail of the Met receptor, as well as Y1003 in the juxtamembrane domain. The 1349/56 phosphorylated tyrosines serve as docking sites for signaling proteins that contain Src homology 2 (SH2) or phosphotyrosine binding (PTB) domains, including Grb2, which recruits the multisubstrate scaffold protein Gab1, whose tyrosine phosphorylation by Met engages multiple downstream pathways and enhances the activation of the Ras/Raf/MEK/ERK signaling pathway as well as the PI3K/Akt signaling pathway (Figure 1). Collectively, these interactions are tightly regulated and ultimately lead to transmitting downstream cellular responses [42,43,44,45,46,47,48,49].

Moreover, the Met receptor can interact with various cell membrane proteins, including integrins, CD44v6 isoform, and plexin-type receptors, as well as interaction with other RTKs, such as EGFR, Her2, Her3, RET, and IGFR1 [46,49,50,51,52,53,54,55]. These interactions enable the engagement of distinct downstream substrates, which leads to subsequent regulation of diverse cellular processes. It is imperative to prioritize the collective efforts to help identify additional Met-interaction hubs and develop a better understanding of Met signaling pathways. A recent study by Salokas et al. used affinity purification coupled with mass spectrometry (AP-MS) to identify stable binding partners and proximity-dependent biotin identification (BioID) to unveil proximal interactions of Met with other proteins. Notably, this identified key interactions between Met and other receptors, including the insulin receptor (INSR), the TYRO3 tyrosine protein kinase receptor, platelet-derived growth factor receptor beta (PDGFR beta), and neurotrophic receptor tyrosine kinase 3 (NTRK3) [56]. These novel interactions with Met reveal new avenues for exploring potential pathways, broadening our understanding of the diverse cellular processes and signaling networks that drive Met signalling in pathophysiological conditions. 

## 3. *MET*/HGF Dysregulation and Oncogenic Paradigms in GBM

**Figure 2:**
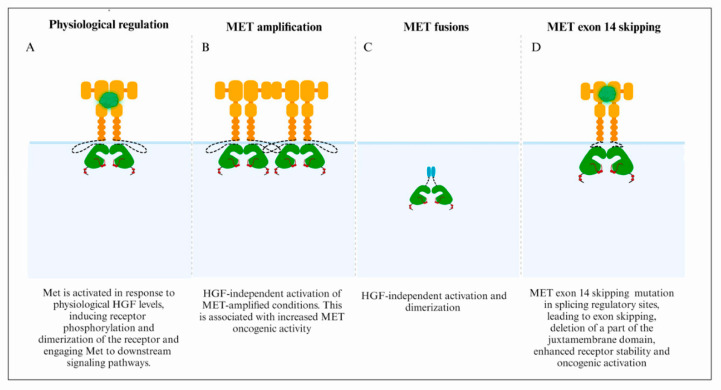
Mechanisms of *MET* oncogenic activation in GBM. (**A**) Physiologically, HGF-METet interaction promotes dimerization and potentially oligomerization of the Met receptor and subsequent trans-phosphorylation of tyrosine residues Y1234 and Y1235 within its kinase domain, subsequently engaging Met with downstream signaling pathways. (**B**) Focal *MET* amplification is associated with high *MET* expression and enhances ligand-independent oncogenic activity. (**C**) Gene rearrangements and chromosomal translocations result in constitutive activation of the fusion proteins that typically self-dimerize in a ligand-independent manner, leading to oncogenic activity. (**D**) Loss of the direct Cbl-TKB-binding site via the loss of exon 14 is associated with reduced ubiquitination, decreased degradation, and sustained Met activation following HGF stimulation, leading to increased oncogenic activity.

*MET* exerts regulatory control over diverse cellular functions, including proliferation, survival, and motility, typically exhibiting low activity in normal cells. However, when *MET* undergoes abnormal activation in tumor cells, it stimulates enhanced growth, angiogenesis, and invasion, leading to adverse overall survival outcomes [57]. Clinical data from various studies, including The Cancer Genome Atlas (TCGA) consortium, indicated the types of *MET* aberrations from the GBM cohort, including focal *MET* amplification, fusion genes, and *MET* exon 14 skipping mutations (Figure 2) [58,59,60,61,62,63]. 

### 3.1. MET Focal Amplification

Focal *MET* amplification is the predominant mechanism of wild-type *MET* alterations in GBM. This amplification of *MET* at chromosome 7 is defined clinically by fluorescent in situ hybridization (FISH), which measures the ratio between the number of *MET* copies and the copies of the chromosome 7 centromere (CEP7). In particular, the cut-off of the *MET*/CEP7 ratio is classified into low (<1.8), medium (1.8 to <4), or high (≥4) [64]. Recent breakthroughs in molecular research have significantly deepened our comprehension of the properties and functions of focal oncogene amplification and rearrangements by introducing innovative methodologies and techniques for their identification, such as copy number variations (CNVs) and whole-genome sequencing (WGS)-based tools [65]. This understanding will help identify the level of amplification and rearrangements, which may predict responses to Met inhibitors and provide an understanding of resistance to other therapeutic options. *MET* amplification is associated with increased Met activity and/ or constitutive kinase activation in the absence of ligand, as observed in higher-grade GBMs with worse clinical outcomes [66,67]. Data from Lal et al. demonstrated that targeting the Met/HGF axis potentiates the response to γ-radiation synergistically, increases apoptosis, and attenuates cell viability in U87 MG human glioma cell lines and glioma xenograft models [68]. These data support the clinical need to evaluate *MET*-targeted therapies in combination with other radiotherapeutic or chemotherapeutic agents. Another independent study by Chi and colleagues showed a rapid and efficient clinical and radiographic response when a GBM patient with confirmed *MET*-amplified status was treated with crizotinib (PF-02341066), a dual ATP competitive inhibitor of Met (cellular IC50, 8 nM) and anaplastic lymphoma kinase (ALK) (cellular IC50, 20 nM) [69]. These data suggest that a subset of GBMs is potentially dependent on *MET* amplification and, therefore, sensitive to *MET* inhibition. Such clinical responses will pave the way to stratifying patients with GBM tumors harbouring *MET* amplification and help advance further clinical investigation on *MET* aberrations as a therapeutic companion target in GBMs. Additional evidence of the involvement of *MET* in the landscape of drug resistance is supported by a study conducted by Min and colleagues, which demonstrated that *MET* signaling activation is essential for GBM stem cells and that METet inhibition suppresses tumor growth and invasiveness [70]. This mechanism was further delineated by De Bacco and colleagues, who identified a subset of radioresistant glioblastoma stem cells (GSCs) driven by the sustained activation of several protein kinases, such as Aurora kinase A, ATM kinase, and the downstream effectors of DNA repair and the phosphorylation and cytoplasmic retention of p21 [71]. The application of Met inhibitors caused DNA damage accumulation in irradiated GSCs and their depletion using in vitro and in vivo xenotransplant models. Regrettably, it is worth noting that the Met inhibitor, JNJ-38877605 used in this study has been precluded from phase 1 clinical trials due to renal toxicity via the formation of species-specific insoluble metabolites [72]. These data suggest that *MET* contributes to GBM cancer stemness and tumour-initiating cells, potentially in a similar manner to its role in a subset of triple-negative breast cancer [73], and is a promising therapeutic target for this disease. 

### 3.2. Fusion Genes 

Chromosomal translocations that result in constitutive activation of RTK are increasingly detected using the latest deep sequencing technologies. *MET* was first identified as an oncogenic fusion between the TPR locus on chromosome 1 and the *MET* intracellular kinase domain on chromosome 7. TPR encodes a leucine zipper domain that constitutively dimerizes the Met kinase domain in the absence of ligand [31]. This fusion protein is cytosolic and constitutively activates downstream signalling pathways [74]. Multiple fusions have been detected in GBM. The *FIG*-*ROS1* RTK fusion was the first gene fusion characterized in GBM [75]. This fusion event was identified as an intra-chromosomal homozygous deletion spanning 240 kilobases on chromosome 6q21. Consequently, extensive evidence has confirmed the constitutive activation of the resultant fusion protein, establishing it as an oncogenic entity. Following this discovery, multiple fusion proteins were identified, many of them involving RTKs [76]. The International Cancer Genome Consortium PedBrain Tumor Project identified *PTPRZ1*-*MET* (ZM) gene fusions in approximately 10% of cases of primary pediatric GBMs [77], whereas another study detected 15% of the ZM fusion gene in secondary GBM cases [78]. To further explore the oncogenic nature of ZM, Huang and colleagues analyzed a larger cohort of 485 glioma patients [79]. This demonstrated that ZM fusions were predominant in lower-grade and secondary GBMs but were not common in primary GBMs. This fusion transcript indicated a worse prognosis in these patients. The PTPRZ1 gene, located on chromosome 7q31.32, encodes the tyrosine phosphatase receptor type Z1 protein, which is closely located to *MET* (location 7q31.2). The fusion of both genes caused by intron insertion and tandem duplication can result in both in-frame and out-of-frame transcripts [78]. Unlike ZM, which contains full-length *MET*, another fusion transcript detected in GBMs is *CLIP2*-*MET*, which maintains only the kinase domain [77]. *MET* fusions result in the upregulation of the mitogen-activated protein kinase (MAPK) signaling pathway, which is associated with aggressive glial tumors in vivo. These tumor formations have been effectively suppressed using Met inhibitors, supporting the advancement of the use of a *MET* inhibitor in the clinic. However, although initial responses to *MET* inhibitors have been observed, the development of resistance can be rapid [80]. To further examine the pathologic role of *MET* fusion genes, Zeng and colleagues characterized exosomes from GBM cells harbouring ZM fusion compared with fusion-free exosomes. This data demonstrated that the internalization of ZM exosomes induced a migratory and invasive phenotype in GBM cells, enhanced neurosphere growth, prompted angiogenesis, and was associated with resistance to temozolomide in GBM cells [81]. Several studies showed inconsistent efficacy when treating pediatric patients with a *MET*-fusion-expressing GBM using crizotinib. Hu et al. characterized a new *MET* inhibitor, PLB-10011, which demonstrated remarkable potency in selectively inhibiting *MET*-altered tumor cells in preclinical models. Molecular dynamic simulation analyses demonstrated that this compound could bind to the conventional ATP-binding pocket of the tyrosine kinase superfamily but with some distinctive interactions in the ATP-binding pocket. An advantage of this small molecule inhibitor is its permeability across the BBB. Subsequently, it has been applied in a phase I clinical trial that enrolled *MET*-altered chemo-resistant glioma patients [82]. In most cases, the N-terminal signal peptide, necessary for protein targeting to the plasma membrane, is deleted in the *MET* fusion genes. This structural rearrangement confers a cytosolic location [31,83]. This cytosolic localization would potentially preclude their entry into the endocytic pathway and, hence, lead to lysosomal degradation, a common pathway for the degradation of cell surface RTKs [84]. The development of genomic and sequencing technologies provides a unique opportunity for systematically characterizing cancer cell transcriptomes, including identifying fusion genes resulting from underlying genomic rearrangements [85]. Consequently, this will pave the way to defining novel therapeutic solutions for GBMs characterized by *MET* gene fusion. 

### 3.3. MET Exon 14 Skipping

Our earlier studies demonstrated that uncoupling the Met receptor from ubiquitination is associated with oncogenic activity in the presence of the HGF ligand, highlighting the significance of negative regulation signals on the Met RTK to suppress its oncogenic activity [86,87]. The intracellular Met juxtamembrane domain is partially encoded by exon 14, which contains Y1003, which, when phosphorylated, is a direct binding site for the TKB (tyrosine kinase binding domain) domain of c-Cbl, an E3 ubiquitin ligase that promotes Met protein ubiquitination and subsequent degradation [86,88]. Consequently, the loss of the direct Cbl TKB-binding site, by loss of exon 14, is associated with reduced ubiquitination, decreased degradation, and sustained Met activation following HGF stimulation, leading to increased oncogenic activity [89,90]. Hundreds of distinct genetic alterations lead to *MET* exon 14 skipping in cancers. These include base substitutions and insertions or deletions (indels) at the splice acceptor site, at the splice donor site, and in intronic noncoding regions that disrupt consensus sequences such as branch sites, polypyrimidine tracts, splice acceptors, and splice donor sites for RNA splicing [91,92,93]. These mutations have been detected in gastric (4.8–7.1%), colorectal (~0–9.3%), and lung adenocarcinoma (3–4%) in addition to GBM. Interestingly, naturally occurring alternative splicing of exon 14 was characterized in cDNA isolated from normal mouse kidney, liver, and brain tissues [94], highlighting that this may also occur in cancers in the absence of mutations.

Recent reports demonstrated that the frequency of METex14del is 14% in secondary GBM, 1% in low-grade GBM (LGG), and 1.7% in primary GBM [82,95,96,97]. The extensive heterogeneity of *MET* genomic alterations leading to exon 14 skipping presents a challenge in clinical practice for routine detection. Although these mutations can be detected by NGS, whole-exome sequencing (WGS), and Sanger sequencing of *MET* exon 14 and its flanking introns [98,99], these are not all applicable for routine clinical testing. A robust, targeted NGS fragment analysis that helps with the systematic identification of patients harboring METex14del mutation has been developed that can be adopted for diagnostic applications in clinical settings [100]. It has been shown that integrating plasma NGS testing into the routine management of cancer patients substantially impacts the detection of therapeutically targetable mutations and improves the design of molecularly guided therapy [101]. Recent reports have also detected *MET* fusion genes coupled with METex14del mutations, which exhibited a poor prognosis [79]. Two types of *MET* TKIs were recently approved for the treatment of lung cancer patients harbouring METex14del: capmatinib (USA) and tepotinib (Japan) [102,103]. Further studies are required to assess the efficacy of these molecules to target *MET* aberrations in GBMs. In this regard, some previous studies and clinical trials evaluated the efficacy of cabozantinib (NCT01639508). This small-molecule tyrosine kinase inhibitor targets multiple tyrosine kinases, including VEGFR2, *MET*, RET, c-KIT, and AXL, and has been clinically evaluated to cross the BBB and has shown clinical efficacy [104,105]. This study addressed the first detailed brain metastases in *MET* exon 14-positive NSCLC and provided preliminary proof of concept of cabozantinib’s efficacy intracranially [104]. 

## 4. *MET*-Targeted Therapies in GBMs

Various therapeutic strategies have emerged to target *MET* oncogenic aberrations in GBMs, including small-molecule inhibitors and *MET*/HGF-specific antibodies. Several clinical trials are studying the efficacy of *MET* TKIs in GBM patients, including cabozantinib (XL-184/BMS-907351) a pan-tyrosine kinase inhibitor for the potential oral treatment of medullary thyroid cancer, multiforme, and NSCLC [106]. This compound has been developed collaboration between Exelixis Inc and Bristol-Myers Squibb Co. This inhibitor has a wide range of targets among the RTK superfamily, including *MET*, *VEGFR-2*, *RET*, *KIT*, *FLT3*, and *TEK*. Fundamentally, this inhibitor is highly potent to Met and exhibits significant oral bioavailability. The potential application of this compound in GBMs is highly attractive in a subset of patients who exhibit hallmarks of angiogenesis [107,108,109]. In addition, this is highly advantageous for targeting the *MET* amplification/overexpression subset of GBMs [24] or suppressing *MET*/HGF autocrine activation in GBM animal models [110]. Cabozantinib was clinically evaluated (phases I and II) in combination with temozolomide and radiation therapy in adult GBM. Another study demonstrated a reasonable clinical response (4.3%) of cabozantinib in patients who had received prior antiangiogenic therapy for GBM [111].

Capmatinib (INC280) is an oral, ATP-competitive, highly potent, and selective Met inhibitor that has a robust interaction mode with Y1230 and a hinge at the ATP-binding pocket [112]. Clinical trials have demonstrated a higher efficacy of capmatinib in a subset of patients with *MET* amplification and METex4del mutation. This data indicates that capmatinib was well tolerated and showed antitumor activity in various solid tumors, including a subset of GBMs [113,114]. However, capmatinib was combined with buparlisib (BKM120), a PI3K inhibitor, and this clinical trial revealed that the combination of capmatinib /buparlisib resulted in reduced exposure of both drugs and a lack of apparent signal/activity in recurrent *PTEN*-deficient GBM [115]. This might be associated with a lack of comprehensive molecular stratification of the patients selected in the study. Another Met inhibitor, HS-10241, which Jiangsu Hansoh Pharmaceutical Co., Ltd., developed, has a very selective and specific inhibitory effect on *MET* and exhibits the ability to pass the BBB, which makes it suitable for targeting *MET* amplification/overexpression in various numbers of solid tumors, including GBMs [116]. This small-molecule inhibitor has been evaluated in clinical trials (phases 1 and II) for individuals with NSCLC harboring METex14del mutations. Volitinib (Savolitinib) is an ATP-competitive inhibitor that has been clinically evaluated in gastric cancer PDX models and showed high anti-tumor efficacy in bearing-amplified *MET*-selected individuals [117]. A phase I clinical trial is being performed in recurrent progressive/refractory high-grade GBM and CNS harboring *MET* aberrations. Recent reports suggest that ALK and its ligand, MIDKINE (MDK), promote the resistance of glioma cells to anticancer therapies such as TMZ [118,119]. Notably, crizotinib can suppress the MDK/ALK axis and effectively enhance the response of glioma-initiating cells (GIC) to TMZ in vitro and using GIC-derived xenograft models [120]. These findings formulate the proof of concept to clinically evaluate the efficacy of crizotinib in combination with TMZ and radiotherapy in newly diagnosed GBMs [121]. 

The utilization of these emerging next-generation pharmacological agents is highly promising when compared with traditional chemotherapy drugs. However, despite the great success of small-molecule-targeted cancer therapeutics, they still face the key challenge of drug resistance [121]. This resistance mechanism in GBM is possibly due to several reasons, including (i) intratumoral and molecular tumor heterogeneity; (ii) hypermutation following alkylating agent treatments; (iii) the Warburg effect, which is characterized by increased aerobic glycolysis and is used to produce ATP for the rapid proliferation of cells associated with higher demands on biosynthetic needs; (iv), tumor immune evasion events; (v) and metabolic reprogramming and activation of distinctive pathways such as glutaminolysis, which has been correlated with the progression of low-grade astrocytoma in an aggressive subtype of GBM [14,122,123,124,125]. Mechanisms of resistance in *MET*-driven tumors include mutations in the small-molecule-binding sites in the *MET* kinase and mutations of other key genes that induce abnormal activation of *MET*, for example, loss of Cbl, upregulation of downstream signaling molecules, or formation of fusion genes [82,83]. Alternatively, monoclonal antibodies are emerging as an effective therapeutic strategy in GBM. The Genentech M monovalent antibody onartuzumab is one of these agents and potently inhibits HGF/Met binding and HGF-dependent Met receptor tyrosine phosphorylation and signaling [126]. Despite the failure of onartuzumab in combination with erlotinib to show efficacy in advanced-stage NSCLC in a phase III clinical trial, it is still a molecule of interest to be studied in combination with bevacizumab, an anti-angiogenic agent, in patients with recurrent GBM [127,128]. Emerging areas include *MET* inhibitor cabozantinib in combination with an immune checkpoint inhibitor, atezolizumab, which is a monoclonal antibody that works by binding to the protein PD-L1 on the surface of some cancer cells [129]. This combination is in phase I/II trials in patients with recurrent GBM (Table 1). 

## 5. Future Directions 

As our understanding of protein structure grows, it becomes possible to predict how and where small molecules, ligands, and/or modulators interact with a protein and identify putative docking sites and the strength/mode of binding [130]. 

A major limitation towards understanding the role of METex14del mutation is the lack of mechanistic studies to delineate the molecular and biochemical structure and functionality of the M receptor with this mutation, including (i) defining the juxtamembrane domain structure to help examine the transitioning of the Met receptor from physiological ligand-dependent (non-amplified) to its pathological state (ligand-independent, amplified). Recent genomic studies have identified METex14del in different types of cancer across diverse populations. However, most of these studies lack stratification into non-amplified METex14del vs. amplified METex14del. This will ultimately help correlate METex14del with other actionable and driver disease mutations. In addition, understanding the juxtamembrane domain structure is very important for developing new strategies that selectively target METex14del downstream signaling. Previous studies have shown that the juxtamembrane domain negatively regulates the kinase domain in other RTKs, such as KIT and RON [131,132]. (ii) Identifying the functionality of the putative phosphorylation sites in the Met juxtamembrane domain is crucial to exploring the key signaling events that this domain promotes, or some distinctive interactions that might negatively regulate the receptor deleting Exon 14 could result in the loss of these phosphorylation sites, leading to an oncogenic outcome. During the preparation of this manuscript, the Jura and Fraser groups employed a deep mutational scanning of the *MET* receptor tyrosine kinase domain that enabled the identification of some conserved motifs that potentially represent a possible mechanism of regulation of Met activity [133]. (iii) Spatial and temporal protein interaction and gene expression analyses are required to help examine the distinctive cellular regulation of METex14del mutation, such as surface localization, internalization, and the identification of interaction networks. 

Computational modeling and the application of artificial intelligence (AI), such as AlphaFold, will help to refine the number of compounds and small molecules in drug discovery, therefore minimizing the lab workload and driving drug discovery forward by reducing the time and cost of bringing new drugs to the market [134].

The development of heterobifunctional small-molecule degraders has seen significant advances recently. One such approach is the use of proteolysis-targeting chimeras (PROTACs). These small molecules consist of two linked moieties, with one binding to the protein of interest and the other recruiting and binding an E3 ubiquitin ligase. This simultaneous binding induces protein ubiquitination, leading to subsequent degradation, followed by the ubiquitin–proteasome system (UPS) [135]. In the 20 years since the first small-molecule PROTAC was reported in the literature by Sakamoto and colleagues [136], massive efforts have been made to advance the optimization of first-generation ROTACs into more drug-like molecules to support in vivo studies and the identification of clinical molecules that function as degraders [137,138,139,140]. This accelerates the development of this technology from academia to the clinic through preclinical and early-phase clinical trials. In 2019 and 2020, clinical trials of PROTACs were established for two cancer targets: estrogen (ER) and androgen (AR) receptors [141,142]. There are several advantages of PROTACs over traditional drug inhibition, including, but not limited to, PROTACs (i) being able to degrade the function of an entire protein and distinguish enzymatic functions of protein kinase compared to the non-enzymatic functions; (ii) being able to overcome drug-resistance mechanisms, which is potentially not impacted by the emergence of mutations the might confer resistance to small-molecule inhibitors; and (iii) having the ability to target undruggable targets [143,144]. Recent studies demonstrated remarkable efficacy in targeting WT and mutant RTKs such as *EGFR* [145,146,147,148], *HER2* [149,150,151], and *MET* [148,152,153]. Although PROTACs have great potency and efficacy to target RTKs, the exact mechanism of how these molecules target these receptors for degradation is unclear. Further exploration of these mechanisms requires additional examination. In this regard, PROTACs are potentially helpful for targeting cytosolic *MET* fusion proteins. Recent reports demonstrated the potential to develop new potent PROTACs that can penetrate the BBB and target indoleamine 2,3-dioxygenase1(IDO1) in GBM [154,155]. This is very promising, as it stresses the development of new degrader molecules targeting *MET* fusion in GBM, which might be a potential therapeutic strategy for selected GBM patients.

## 6. Conclusions

In this review, we highlighted the fundamental role of Met signaling in health and disease, focusing on the oncogenic mechanisms of activation in GBM. Recently, genomic technologies facilitated the discovery of some *MET* aberrations, such as *MET* fusion genes and METex14del mutation, which are highly beneficial to identifying patients who would benefit from specific treatments. Diagnostic assays in clinical settings should be improved to capture these genomic signatures and facilitate the biomarker discovery process and the development of point-of-care assays to help with early, precise, and accurate disease detection. AI enhances the drug discovery process and will ultimately define new structural insights of Met and assist in identifying the development of new therapeutic options for patients with *MET*-dysregulated cancers.

## Figures and Tables

**Table 1:** List of *MET*-targeted therapies in the treatment of *MET* aberrations in glioblastoma in clinical trials.

Study NCT Number	Summary	Conditions	Drug(s)	Mechanisms of Action	Phase
00704288	Evaluate XL184 (cabozantinib) for GBM	Glioblastoma multiforme	Drug: XL184	VEGFR2, *MET*, and RET inhibitor	II [104] completed
00960492	Determine safe dose of XL184 (cabozantinib)	Glioblastoma	Drug: XL184, temozolomide, radiation therapy	VEGFR2, *MET*, and RET kinase inhibitor	I [111] completed
01068782	Evaluate XL184 (cabozantinib) in astrocytic tumors	Grrade IV astrocytic tumors	Drug: XL184	VEGFR2, *MET*, and RET kinase inhibitor	II completed
01324479	Evaluate INC280 (capmatinib) for solid tumors	Solid tumors	Drug: INC280	*MET* inhibitor	I [114] completed
01870726	Evaluate INC280 (capmatinib) and buparlisib for recurrent glioblastoma	c-*MET* inhibitor, PI3K inhibitor, PTEN mutations, homozygous del. of PTEN or PTEN neg. by IHC, c-Met amplification by FISH, INC280, BKM120, buparlisib, recurrent GBM	DrugRUG: INC280, buparlisib	*MET* and PI3K inhibitor	I/II [115] active
02386826	Evaluate INC280 (capmatinib) and bevacizumab for recurrent glioblastoma	Glioblastoma gliosarcoma	Drug: INC280, bevacizumab	*MET* and VEGFR inhibitor	I completed
01441388	A study of crizotinib plus VEGF inhibitor combinations	Advanced solid tumors	Drug: crizotinib plus VEGF inhibitor combinations	*MET* and VEGFR inhibitor	I completed
02270034	Assess crizotinib for newly diagnosed glioblastoma	Glioblastoma multiforme (grade IV) of cerebellum	Drug: crizotinib	*MET* inhibitor	I completed
01632228	Assess onartuzumab and bevacizumab for recurrent glioblastoma	Glioblastoma	Drug: bevacizumab, onartuzumab, placebo	*MET* and VEGFR inhibitor	I [127] completed
02885324	Study cabozantinib for CNS tumors in children	Glioblastoma multiforme, anaplastic astrocytoma, malignant brain tumor, high-grade glioma	Drug: cabozantinib	VEGFR2, *MET*, and RET inhibitor	II completed
03175224	Study APL-101 for advanced solid tumors	Solid tumor, advanced cancer, renal cancer, gastric cancer, gastroesophageal junction adenocarcinoma, NSCLC, lung cancer, brain tumor, glioblastoma multiforme	D: APL-101 oral capsules	*MET* inhibitor	I/II active
03598244	Assess volitinib (savolitinib) for recurrent primary CNS tumors	Central nervous system (CNS) tumors	Drug: volitinib (savolitinib)	*MET* inhibitor	I active
05039281	Study atezolizumab and cabozantinib for recurrent glioblastoma	Recurrent glioblastoma, recurrent gliosarcoma	Biological: atezolizumab, Drug: cabozantinib	Atezolizumab: targets PD-L1, cabozantinib: VEGFR2, *MET*, and RET kinase inhibitor	I/II active
